# The effects of football juggling learning on executive function and brain functional connectivity

**DOI:** 10.3389/fnhum.2024.1362418

**Published:** 2024-03-07

**Authors:** Xiaoxiao Dong, Xiang Gui, Sebastian Klich, Lina Zhu, Dandan Chen, Zhiyuan Sun, Yifan Shi, Aiguo Chen

**Affiliations:** ^1^Nanjing Sport Institute, Nanjing, China; ^2^Yangzhou University, Yangzhou, China; ^3^Department of Paralympic Sport, Wroclaw University of Health and Sport Sciences, Wroclaw, Poland

**Keywords:** football juggling, motor learning, executive function, functional connectivity, functional magnetic resonance imaging

## Abstract

This study aimed to explore the relationship between motor skill learning and executive function (EF), with an emphasis on the potential effects of football juggling learning. A randomized controlled trial involving 111 participants aged 17–19 years was conducted. Participants were randomly assigned to either the football juggling learning (FJL) group or a control group. The FJL group underwent 70 sessions of football juggling learning, while the control group engaged in their normal daily activities without any exercise intervention during the same time frame. Both groups were assessed for EF performance and underwent functional magnetic resonance imaging (fMRI) scans before and after the experiment. The executive function test included three tasks, namely, inhibition, working memory, and shifting. The results showed significant improvement in inhibition and shifting in both groups, and the FJL group showed greater improvement in these aspects of EF compared to the control group. Additionally, in comparison to the control group, the FJL group exhibited increased functional connectivity within the frontal, temporal, and cerebellar regions from the pre-test to the post-test. Notably, enhanced functional connectivity between the right superior temporal gyrus (posterior division) and left cerebellum 6 was identified in the FJL group and was associated with improved EF performance induced by football juggling learning. These findings shed light on the potential causal relationship between motor skill learning, EF, and brain plasticity. Importantly, our study provides preliminary evidence supporting the use of motor skill learning, such as football juggling, as a potential avenue for cognitive enhancement.

## 1 Introduction

The acquisition of new motor skills remains a continuous challenge and opportunity for adaptation throughout life. This ongoing process facilitates environmental acclimation and empowers individuals to compensate for potential impairments or injuries. Motor skill learning, specifically, is a dynamic process that unfolds over time. The acquisition of motor skills requires repeated practice, during which a substantial allocation of cognitive resources, such as attention, planning, and working memory, is utilized for the execution of movements (Diamond, 2015; Alesi et al., 2016; Formenti et al., 2021). Researchers propose that this learning process can enhance cognitive development, particularly in the realm of executive functions (Diamond, 2015).

Executive function is broadly defined as a constellation of “higher-order cognitive processes” that orchestrate and govern more fundamental cognitive mechanisms (Funahashi, 2001). The core components of executive function typically include inhibition, working memory, and shifting (Funahashi, 2001; Friedman and Miyake, 2017). Inhibition encompasses the ability to deliberately suppress dominant, automatic, or prepotent responses when required. Working memory, described as a multifaceted system, not only allows for temporary active storage of information but also facilitates its manipulation and processing. Shifting, as the term suggests, is the dynamic process of transitioning between multiple tasks, operations, or mental sets (Miyake et al., 2000). Cross-sectional research employing the expert-novice paradigm offers compelling evidence supporting the notion that motor skill learning fosters the development of executive functions (Kida et al., 2005; Chan et al., 2011; Moratal et al., 2020). For example, Kida et al. (2005) demonstrated significantly faster Go/Nogo reaction times in baseball players compared to non-athletes. Verburgh et al. (2014) reported superior motor inhibition in highly skilled soccer players, as evidenced by shorter stop signal reaction times and a larger alerting effect on the attention network test. Further exploration by Ishihara et al. (2017a,b) revealed a positive association between tennis frequency and enhanced processing speed, inhibitory control, and working memory in children and adolescents. Furthermore, a longitudinal study examining bicycle learning demonstrated a significant relationship between the acquisition of motor skills and the improvement of executive function, emphasizing the importance of cognitively engaging exercise for optimizing executive function (Tse et al., 2021).

Understanding the intricate relationship between motor skill learning and executive function has captivated researchers for years. Over the years, significant emphasis has been placed on the neural mechanisms underlying this association, seeking to elucidate the impact of motor skill acquisition on cognitive development (Diamond, 2000; Ito, 2008; Leisman et al., 2016). One compelling perspective posits a functional coupling between motor and cognitive processes, echoing their shared evolutionary origins (Leisman et al., 2016). Notably, both cognitive and motor functions are orchestrated by a consortium of brain regions, including the prefrontal cortex, cerebellum, and basal ganglia. These interconnected areas collaborate to regulate and control the execution of both cognitive tasks and physical activity (Chayer and Freedman, 2001; Ito, 2006). Yang et al. (2020) conducted a comprehensive analysis comparing whole-brain functional connectivity and attention in fast-ball sports athletes and non-athletes. Their findings revealed significantly increased neural efficiency in specific brain regions of athletes compared to non-athletes, with this enhancement directly linked to improved attention-motor modulation and executive control. While several studies have shed light on the potential neural mechanisms by which motor skill learning reinforces EF, most existing research relies on cross-sectional designs. This methodological limitation leaves unanswered questions regarding the causal relationship between motor skill learning, EF, and brain plasticity. To bridge this gap, longitudinal randomized controlled intervention studies are crucial. Such investigations can examine the causal relationship between motor skill learning, EF, and the brain, as well as explore the neural mechanisms underlying the promotion of EF development through the learning of motor skills.

In the present randomized controlled trial, we employed football juggling as an instructional task. Football juggling is a complex learning task requiring multimodal skills, involving more limb motor activity dependent on multi-sensory feedback. Indeed, the initial stages of football juggling acquisition necessitate substantial cognitive engagement, particularly in the domains of attention, control, and memory. This learning process has the potential to facilitate the development of executive functions. To delve deeper into the neural mechanisms underpinning the enhancement of executive function development through motor skill acquisition, we employed resting-state functional magnetic resonance imaging (rs-fMRI) to analyze resting-state functional connectivity (Rs-FC). Prior research exploring the relationship between motor skill learning and the brain has largely concentrated on alterations in brain structure, with investigations of functional changes being comparatively limited (Draganski et al., 2004, 2006; Wenger et al., 2017; Zhang et al., 2021). Rs-FC refers to the temporally correlated, low-frequency fluctuations in blood oxygen level-dependent (BOLD) signals across brain regions observed during periods of non-task engagement (Biswal, 2012; Buckner et al., 2013). These temporal correlations are not random, exhibiting consistent patterns across studies and participants. Furthermore, there is growing consensus regarding the behavioral significance of the strength of correlations within and between brain networks. Notably, rs-fMRI has been proposed as an effective measure of brain plasticity, with resting-state activity patterns reflecting the history of synchronized activation between brain regions. Consequently, exploring changes in rs-FC can contribute to a deeper understanding of how motor skill learning alters connectivity patterns. This bears significant importance for achieving a comprehensive understanding of the neural mechanisms underlying the promotion of executive function development through motor skill learning.

This longitudinal study explores the impact of football juggling on executive function and its underlying neural mechanisms, employing rs-FC analysis. By investigating the causal relationship between motor skill learning, EF, and the brain, this research aims to provide direct evidence for this crucial brain-behavior link. This study rests upon the following three guiding assumptions: first, that learning to juggle for 70 sessions enhances EF performance; second, that juggling effectively increases brain functional connectivity; and finally, that enhanced functional connectivity may be related to the improvement in EF observed after learning football juggling.

## 2 Materials and methods

### 2.1 Participants

A total of 111 student participants were recruited for the study by distributing advertisements across the campus. Table 1 details their demographic and physical fitness characteristics. To be eligible for participation in the experiment, individuals had to satisfy the following criteria: possess normal vision (or corrected-to-normal), be right-handed, have no history of mental disorders or psychoactive substance use, possess no prior experience with soccer training, and meet the requirements for magnetic resonance imaging (MRI), including the absence of implanted metal devices (e.g., dentures) and the non-use of body-borne electronic, magnetic, or mechanical devices (e.g., pacemakers).

**Table 1:** Demographic data and physical fitness (mean ± SD).

**Variables**	**Football juggling learning group**	**Control group**	***P*-value**
*N*	38	32	–
Sex (male/female)	(27/11)	(20/12)	0.45
Age (years)	18.34 ± 0.58	18.34 ± 0.48	0.99
BMI	20.23 ± 2.89	20.72 ± 2.60	0.46
Flexibility (cm)	13.04 ± 6.46	13.04 ± 6.71	0.99
Speed-of-movement (s)	7.84 ± 0.69	7.73 ± 0.86	0.57
Strength (m)	1.97 ± 0.28	2.03 ± 0.40	0.52

Participants were randomly assigned to either the football juggling learning group (*n* = 68; 23 female and 45 male participants) or the control group (*n* = 43; 14 female and 29 male participants). A total of 111 participants initially participated in the experiment. However, 41 participants were excluded for the following reasons. 1. Not fulfilling the criteria for juggling: eleven participants were excluded from the juggling group for failing to reach a minimum of 35 juggles. 2. Injury: three participants were excluded due to injuries sustained during the intervention. 3. Missing fMRI data: eleven participants were excluded due to missing pre- or post-test magnetic resonance imaging data. 4. Data quality issues: sixteen participants were labeled “>20% of frames labeled as scrubbed”, indicating poor image quality, so their imaging data was excluded. Therefore, 70 participants were included in the final analysis: 38 in the football juggling learning group and 32 in the control group.

The study was given ethical approval by the Affiliated Hospital of Yangzhou University (2017-YKL045-01), and written informed approval was obtained from all participants before the experiment.

Prior to conducting our study, a statistical power analysis was performed to determine the necessary sample size for a repeated-measures ANOVA. This analysis employed a between-subjects factor (football juggling learning group vs. control group) and a within-subjects factor (post-test vs. pre-test). The parameters specified for the analysis were (1) a medium effect size (Cohen's *d* = 0.25), (2) a power of 0.80, and (3) an alpha level of 0.05. This analysis indicated that a sample size of 46 participants (23 in each group) would be sufficient to achieve statistical significance. To account for potential participant dropouts during the experiment period, the sample size was subsequently increased to 111 participants.

### 2.2 Experiment procedure

In preparation for the study, participants completed physical quality tests and provided basic demographic information 1 week prior to the baseline scan and executive function assessment. During the baseline period, all participants underwent an fMRI scan and an executive function test, with the latter being preceded by a confirmation of task understanding by the experimenter. Following baseline data collection, participants were randomly assigned to either the football juggling learning group or the control group. The football juggling learning group engaged in 70 sessions of 30-min football juggling learning, spread over 82 days, with teacher supervision to ensure attendance and non-disruptive learning. Participants could suspend practice temporarily, but for no more than two consecutive days, to ensure learning occurred without disruption (Chen et al., 2021). The second test, including another scan and executive function assessment, was conducted for the football juggling learning group after completing the 70 sessions, while the control group underwent the same test after 70 days of no juggling learning. Upon experiment completion, both participants and their legal guardians received fair compensation for their involvement.

### 2.3 Football juggling learning

Prior to the learning task, participants were informed of the mandatory requirement to complete 70 sessions of football juggling. Failure to meet this prerequisite resulted in exclusion from the task. This research, drawing upon the core tenets of traditional football course instruction, independently designed and deployed instructional videos dedicated to football juggling. The teaching methodology involved organizing learners to watch these instructional videos. After video viewing, self-study and practice were undertaken, guided by the video's exercise methods and key movements. Throughout the learning process, the instructional videos remained readily accessible for review at any time. The whole process is conducted by a football teacher, who uses a unified teaching language to provide learners with error correction and guidance. In total, the participants underwent 70 sessions of football juggling learning, each lasting 30 min at a fixed location once a day. The selection of 70 sessions for football juggling learning was based on the collective teaching experience of football instructors and preliminary experimental findings.

### 2.4 Behavioral test

#### 2.4.1 Football juggling test

Upon the conclusion of the learning period, participants were subjected to a football juggling test. The test objective, established in accordance with the student's football skills assessment criteria issued by the Ministry of Education of the People's Republic of China (http://www.moe.gov.cn), was the continuous juggling of 35 kicks within a 60-s timeframe. Participants deemed sufficiently skilled were those who successfully achieved this benchmark. The test was administered twice, with the best score of the two attempts recorded as the participant's performance.

#### 2.4.2 Physical fitness test

To account for the influence of physical fitness on football juggling learning, participants underwent a battery of physical fitness tests based on the Chinese National Student Physical Fitness Standard (CNSPFS). This assessment employed three distinct components: strength, speed of movement, and flexibility. The standing long jump test (m) evaluated strength fitness, while the 50-m sprint run test (s) assessed speed-of-movement fitness. Finally, the sit-and-reach test (cm) measured flexibility and fitness.

#### 2.4.3 EF test

The test tool designed by Chen et al. (2014) was employed to evaluate the participant's executive function. Three computer-based neuropsychological tasks assessed different aspects of EF: inhibition, working memory, and shifting.

Inhibition was measured using a modified flanker task. English letters were presented on the screen and categorized into congruent trials (“XXFXX” or “XXLXX”) and incongruent trials (“LLFLL” or “FFLFF”). Participants responded by pressing the “F” key when the middle letter was “F” and the “L” key when the middle letter was “L”. Both conditions appeared equally often and were presented in a randomized order. To assess behavioral performance, both response accuracy (ACC) and reaction time (RT) were recorded for both congruent and incongruent trials (Eriksen and Eriksen, 1974). Higher ACC and shorter RT indicated better performance.

A 2-back task was employed to assess participants' working memory. This task involved rapidly presenting a sequence of letters (e.g., B, D, L, Y, and O) in the center of the screen. Participants were instructed to judge whether the current letter matched the one presented two positions back. If it did, they pressed the “F” key; if it did not, they pressed the “L” key. Accuracy and reaction time for correct trials were averaged as the main behavioral index, with higher ACC and shorter RT indicating better performance.

To investigate executive function in terms of shifting, a more odd task was utilized (Salthouse et al., 1998; Hillman et al., 2006). This task comprised three parts: A, B, and C. Each part displayed a series of numbers (1–4 or 6–9) in the center of the screen, requiring participants to make different judgments based on specific conditions. Part A, the homogeneous black condition, involved judging whether the number was greater than or less than 5. The “D” key was pressed for >5, and the “F” key for <5. Part B, the homogeneous green condition, involved judging whether the number was odd or even. The “J” key was pressed for odd, and the “K” key for even. Part C, the heterogeneous condition, combined A and B, alternating presentation every two trials. Black numbers >5 required “D”, black numbers <5 required “F”, green odd numbers required “J”, and green even numbers required “K”. Response accuracy and reaction time were collected in both homogeneous and heterogeneous conditions to assess behavioral performance. Higher ACC and shorter RT indicated better performance. Specific details of these three tasks have been previously reported (Chen et al., 2011). Stimulus presentation and response data collection were conducted using the E-Prime 1.1 software (Psychology Software Tools Inc., Pittsburgh, United States).

### 2.5 Functional MRI data acquisition and image pre-processing

#### 2.5.1 MRI data acquisition

Participants underwent three scans for high-resolution structural images of the whole brain on a 3.0T GE Discovery MR750W scanner in the Affiliated Hospital of Yangzhou University. Before each scan, the researchers used the same vacuum pillow and/or foam padding to hold the head position in a stable manner. An anatomical T1-weighted MRI was acquired using a gradient-echo sequence with the following parameters: acquisition matrix = 256 × 256, repetition time (TR) = 7.20 ms, echo time (TE) = 3.06 ms, field of view (FOV) = 256 × 256 mm, flip angle (FA) = 12°, slice thickness = 1.0 mm, T1 = 450 ms. The rs-FC fMRI data was collected using an echo-planar imaging sequence with the following parameters: acquisition matrix = 256 × 256, TR = 2,000 ms, TE = 30 ms, FOV = 224 × 224 mm, FA = 90°, slice thickness = 4.0 mm.

#### 2.5.2 Image pre-processing

The rs-FC data underwent comprehensive preprocessing and analysis within the Statistical Parametric Mapping (SPM12) and CONN toolbox (version 18b) (https://www.nitrc.org/projects/conn/). Preprocessing steps involved: realignment of functional runs and motion correction, co-registration of functional and anatomical images per participant, normalization to the Montreal Neurological Institute template, and spatial smoothing with a 6-mm FWHM Gaussian filter. To further control for motion artifacts, the Artifact Detection Toolbox (ART) with conservative settings (95th percentile threshold and composite subject motion > 0.5 mm) identified outlier time points (global signal Z > 3) for scrubbing. Participants exceeding 20% flagged frames were excluded. The identified outliers were then modeled as nuisance regressors for volume censoring. Additional denoising in CONN utilized the aCompCor method to extract five white matter and five cerebrospinal fluid components, along with realignment parameters. Finally, band-pass filtering (0.01–0.1 Hz) refined the data by reducing noise and enhancing sensitivity, as described by Whitfield-Gabrieli and Nieto-Castanon (2012).

### 2.6 Statistical analysis

The researchers employed Statistical Package for the Social Sciences (SPSS version 24) for all behavioral analyses. To ensure group equivalence, independent sample *t*-tests were conducted for continuous demographic and fitness variables, while χ^2^ tests assessed sex distribution. Statistical significance was set at *p* ≤ 0.05, with Cohen's d reported for effect size. Data normality and variance equality were verified using the Shapiro-Wilk and Levene's tests, respectively. Subsequently, a two-way RM-ANOVA with repeated measures (time: pre-test vs. post-test and group: football juggling learning vs. controls) was conducted for executive function performance, including simple effect analyses.

To explore functional connectivity changes between groups, region-of-interest (ROI)-to-ROI analysis was employed, which is a common approach in fMRI connectivity research. ROIs were derived from the Harvard-Oxford and AAL Atlases, encompassing cortical and subcortical areas from the former and cerebellar areas from the latter. Residual BOLD time courses were extracted from 132 ROIs across the whole brain for each subject, and correlation coefficients were calculated and then transformed to normally distributed scores using Fisher's transformation. To detect group differences in connectivity, a 2 × 2 mixed ANOVA interaction test was performed for each ROI-ROI pair, with significant results reported at *p* < 0.05 after false discovery rate (FDR) correction (Chumbley et al., 2010). Finally, for the football juggling learning group, Pearson correlation coefficients (*r*) were calculated between EF performance changes and functional connectivity changes for each reported ROI-ROI pair, with *p* ≤ 0.05 indicating statistical significance.

## 3 Results

### 3.1 Demographic, physical fitness, and learning performance data

As shown in Table 1, at baseline, the experimental and control groups exhibited no statistically significant differences in demographic or physical fitness characteristics. This was confirmed by independent *t*-tests, which revealed no significant between-group variations in gender distribution (chi-square = 0.58, *p* > 0.05), age [*t*_(68)_ = −0.01, *p* > 0.05], body mass index (BMI) [*t*_(68)_ = −0.74, *p* > 0.05], strength [*t*_(68)_ = −0.64, *p* > 0.05], speed-of-movement [*t*_(68)_ = 0.57, *p* > 0.05], or flexibility [*t*_(68)_ = 0.01, *p* > 0.05]. Notably, the FTL group participants successfully completed a mean of 43.86 ± 11.69 football juggling attempts.

### 3.2 EF performance

Table 2 presents the baseline (pre-test) and post-test mean ± SD for EF performance across inhibition, working memory, and shifting. Initial group comparisons revealed no significant differences in any measured characteristics.

**Table 2:** Performance results for EF, segregated by time point.

	**Football juggling learning group**		**Control group**	
	**Pre-test**	**Post-test**	**Pre-test**	**Post-test**
**ACC (%)**				
**Inhibition**				
Congruent	96.98 ± 3.74	97.20 ± 2.96	95.90 ± 3.49	97.36 ± 2.48
Incongruent	96.55 ± 2.58	95.56 ± 3.47	96.81 ± 2.05	94.28 ± 4.33
**Working memory**	84.52 ± 10.43	90.39 ± 8.28	79.49 ± 12.04	89.40 ± 11.20
**Shifting**				
Homogeneous	95.89 ± 4.83	95.89 ± 3.57	94.87 ± 5.62	96.18 ± 2.71
Heterogeneous	90.68 ± 7.73	94.94 ± 3.68	90.33 ± 5.65	94.14 ± 4.59
**RT (MS)**				
**Inhibition**				
Congruent	495.25 ± 41.37	460.55 ± 20.99	478.86 ± 42.29	458.12 ± 25.42
Incongruent	504.82 ± 35.12	471.46 ± 29.47	491.38 ± 43.32	478.75 ± 31.07
**Working memory**	1,113.27 ± 171.34	912.94 ± 191.77	1,110.44 ± 186.40	897.45 ± 159.34
**Shifting**				
Homogeneous	587.86 ± 65.30	533.45 ± 40.94	570.64 ± 63.92	550.15 ± 43.98
Heterogeneous	961.08 ± 156.97	792.83 ± 80.39	899.00 ± 127.72	795.64 ± 94.78

**Figure 1:**
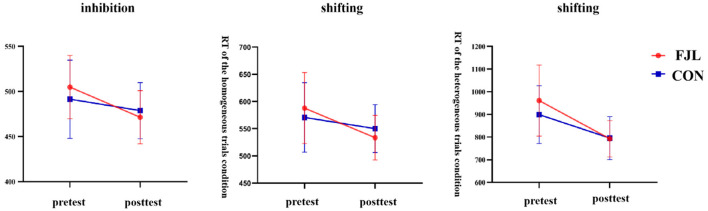
The impact of football juggling learning on inhibition and shifting. FJL, football juggling learning group; CON, control group.

For inhibition, a two-way RM-ANOVA demonstrated a significant interaction effect for RT on incongruent trials (*F*_1,68_ = 9.57, *p* < 0.01, η^2^ = 0.12). Subsequent follow-up analysis revealed non-significant pre-test (*F*_1,68_ = 2.06, *p* > 0.05) and post-test (*F*_1,68_ = 1.01, *p* > 0.05) differences between the experimental and control groups. The control (*F*_1,68_ = 6.54, *p* < 0.05) and FTL groups (*F*_1,68_ = 54.20, *p* < 0.001) both showed significant improvement from pre- to post-test. We found that the RT of the experiment group was higher than the control group in the pretest. For the post-test, the experiment group was lower than the control group (Figure 1).

Similar patterns were observed for shifting. A significant interaction effect for RT on homogeneous trials (*F*_1,68_ = 8.50, *p* < 0.01, η^2^ = 0.11) was identified, with no significant pre-test (*F*_1,68_ = 1.23, *p* > 0.05) and post-test (*F*_1,68_ = 2.70, *p* > 0.05) group differences. Both groups displayed significant pre-to-post improvements, with the FTL group exhibiting a larger improvement (*F*_1,68_ = 47.83, *p* < 0.001) compared to the control group (*F*_1,68_ = 5.71, *p* < 0.05). Consistently, the FTL group outperformed the control group in the post-test with a lower RT, even though the differences between groups were not significant. The heterogeneous trial condition also revealed a significant interaction effect for RT (*F*_1,68_ = 5.49, *p* < 0.05, η^2^ = 0.08). While no significant pretest (*F*_1,68_ = 3.21, *p* > 0.05) and posttest (*F*_1,68_ = 0.02, *p* > 0.05) differences were observed, both groups displayed substantial pre-to-post improvements, with the FTL group showing significantly greater improvement (*F*_1,68_ = 80.77, *p* < 0.001) compared to the control group (*F*_1,68_ = 25.67, *p* < 0.001). Similar to the first two results, the higher RT in the FTL group was reversed in the post-test compared to the control group (Figure 1).

Overall, these findings suggest that football juggling learning improved EF performance across inhibition and shifting to a greater extent than not participating in any activity between the pre-test and post-test.

### 3.3 Functional connectivity

**Figure 2:**
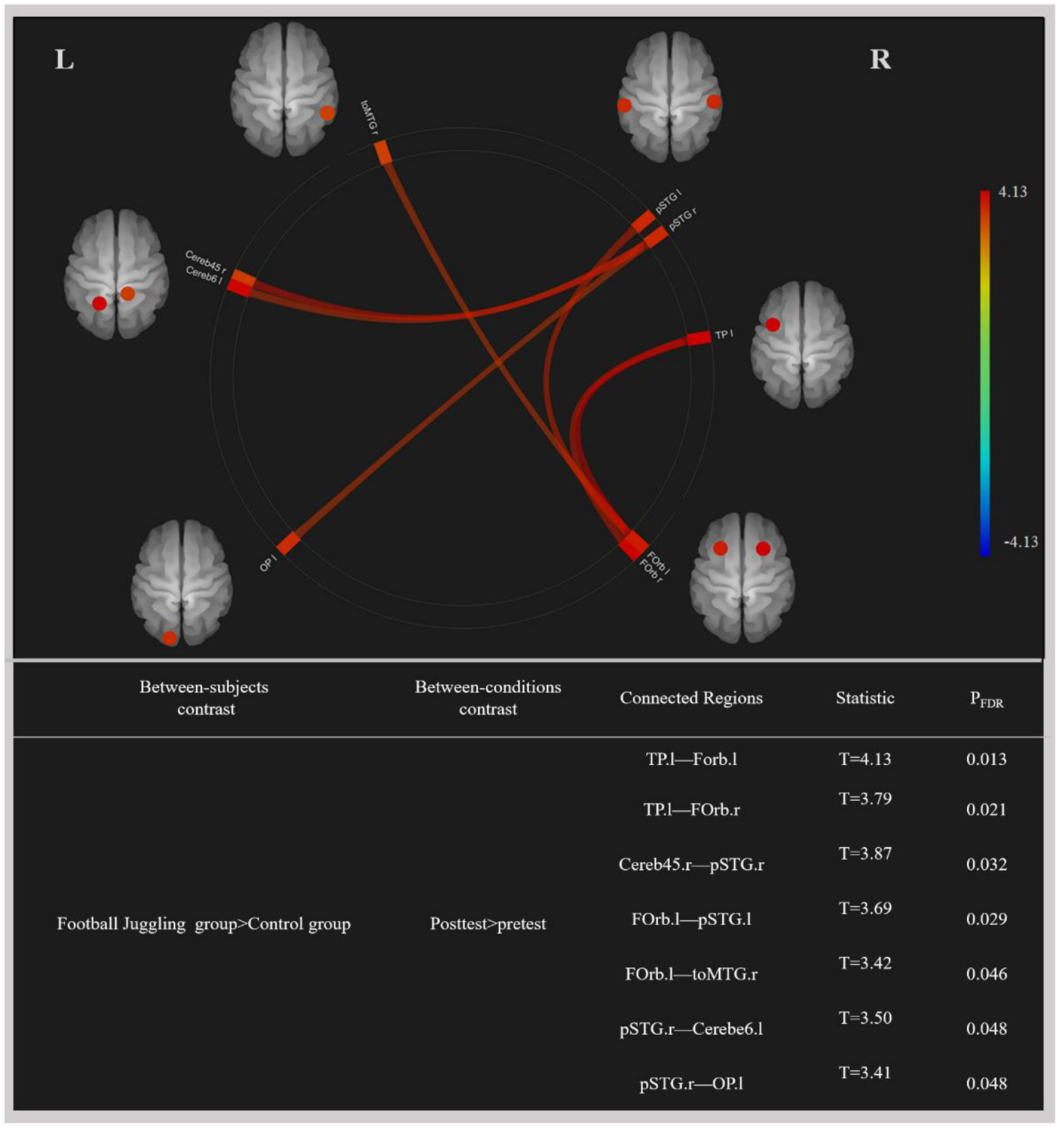
Changes in functional connectivity by football motor learning. Color map: red indicates increased functional connectivity reflected by increased blood-oxygen-level-dependent signal time series synchronization, and blue indicates decreased functional connectivity reflected by decreased blood-oxygen-level-dependent signal time series synchronization (all *p* < 0.05, corrected for multiple comparisons with the FDR approach). OP.l, left occipital pole; Forb.l, left frontal orbital cortex; Forb.r, right frontal orbital cortex; TP.l, left temporal pole; pSTG.l, left superior temporal gyrus, posterior division; pSTG.r, right superior temporal gyrus, posterior division; toMTG.r, right middle temporal gyrus, temporooccipital part; Cerebe6.l, left cerebellum 6; Cereb45.r, right cerebellum 4 5; L, left; R, right.

A 2 × 2 mixed ANOVA interaction test identified statistically significant differences in functional connectivity between the FTL and control groups (*P*_*FDR*_ < 0.05), as depicted in Figure 2. In the FTL group compared to the control group, we observed enhanced functional connectivity between (a) the left temporal pole (TP.l) and left frontal orbital cortex (FOrb.l), (b) the left temporal pole (TP.l) and right frontal orbital cortex (FOrb.r), (c) the right cerebellum 4 5 (Cereb45.r), right superior temporal gyrus, and posterior division (pSTG.R), (d) the left frontal orbital cortex (FOrb.l), left superior temporal gyrus, and posterior division (pSTG.l), (e) the left frontal orbital cortex (FOrb.l), right middle temporal gyrus, and temporooccipital part (toMTG.r), (f) the right superior temporal gyrus, posterior division (pSTG.r), and left cerebellum 6 (Cerebe6.l), and (g) the right superior temporal gyrus, posterior division (pSTG.r), and left occipital pole (OP.l).

### 3.4 Correlations between functional connectivity and EF performance

**Figure 3:**
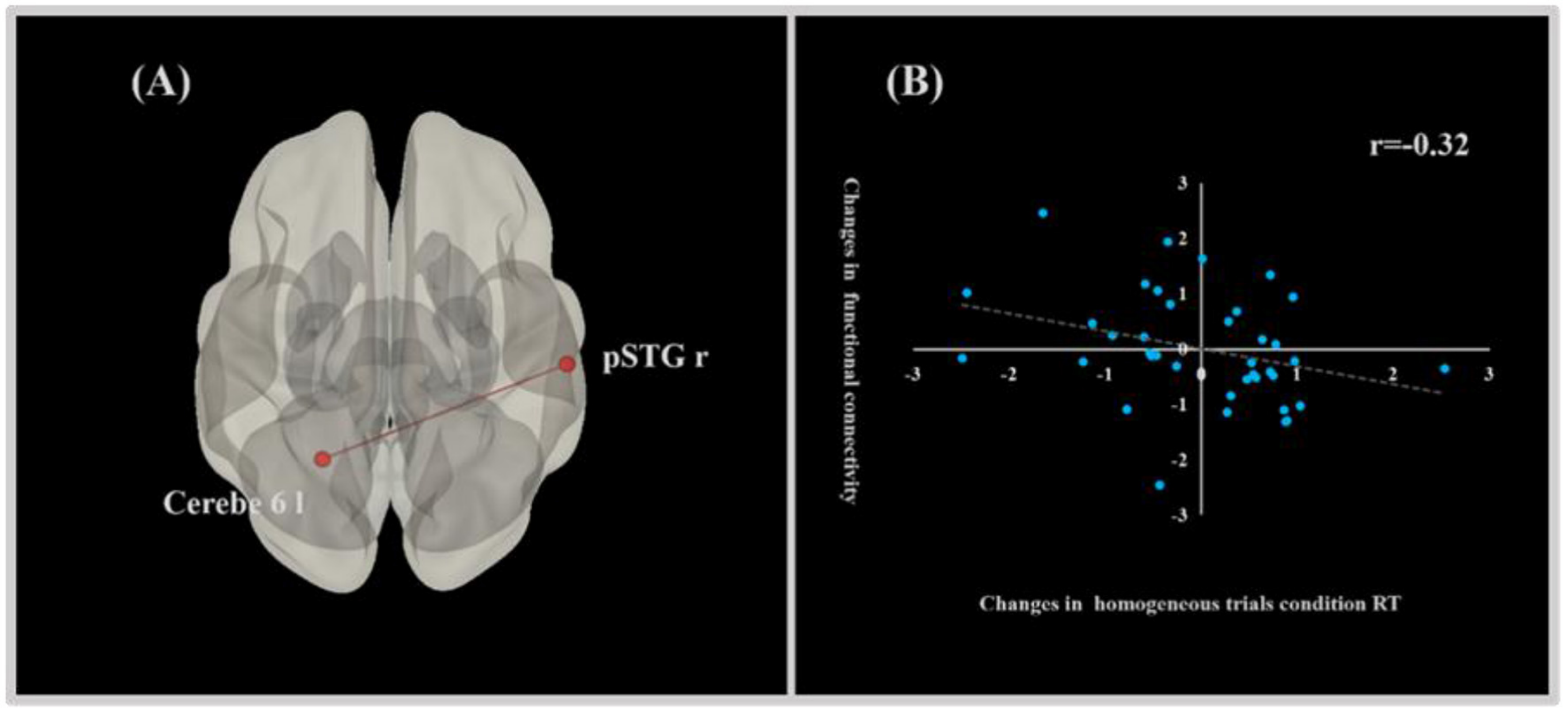
Correlation graph of changes in behavioral performance of EF and functional connectivity. **(A)** Sketch map of functional connectivity related to the behavioral performance of EF. **(B)** Correlation graph of changes in the behavioral performance of EF and functional connectivity.

There was a significant relationship between functional connectivity and EF performance in the FTL group. Specifically, enhanced functional connectivity from the right superior temporal gyrus, posterior division, to the left cerebellum 6, observed from pre-test to post-test, was significantly associated with decreased RT in the homogeneous trials condition of the shifting task (*r* = −0.32, *p* ≤ 0.05). This finding suggests that strengthened functional connectivity between these brain regions is thought to be the underlying neural mechanism for the improved performance of shifting in the FJL group (Figure 3).

## 4 Discussion

Our investigation into the impact of motor skill learning on EF and functional connectivity in college students yielded three key findings aligning with our hypotheses. First, engaging in 70 sessions of football juggling demonstrably enhanced EF performance, particularly in the domains of inhibition and shifting, although we also saw enhanced EF performance in the control group. Second, this same intervention effectively strengthened brain functional connectivity, predominantly within the temporal, frontal, and cerebellar regions. Third, a notable correlation emerged between the increased functional connectivity and the improvement in shifting performance.

Our findings demonstrate that football juggling learning can enhance specific aspects of EF, namely inhibition and shifting. This aligns with existing research highlighting the ability of motor skill learning to improve EF (Wang et al., 2013; Formenti et al., 2021; Koch and Krenn, 2021). However, our results did not reveal any significant benefits for working memory. This discrepancy may be explained by the Fitts and Posner three-stage learning model, which suggests that novel motor skill acquisition progresses through cognitive, associative, and autonomous stages (Fitts and Posner, 1967). The cognitive stage, characterized by high cognitive resource demands, necessitates significant attention, inhibition, and flexibility to execute specific action sequences and achieve desired outcomes (Magill and Anderson, 2021). Given the complexity of football juggling and its reliance on multi-modal skills, including limb motor activity contingent on multi-sensory feedback, achieving sustained juggling likely required participants to maintain high levels of attention to regulate and control their body movements during repeated practice. This sustained focus on inhibition and shifting may have led to enhancements in these aspects of EF within the FJL group. In contrast, the relatively simple action sequences involved in juggling may not have strongly engaged participants' working memory, potentially leading to the observed lack of improvement in this aspect of EF.

Moreover, the football juggling learning group demonstrated enhanced functional connectivity compared to the control group, with increased connections observed between various brain regions crucial for motor skill learning. Notably, strengthened functional connectivity was found in the left superior temporal gyrus to both the left and right inferior frontal gyri, the right cerebellum 4–5 to the right posterior superior temporal gyrus, the left FOrb to both the left pSTG and right temporo-parietal junction, and the right pSTG to both the left cerebellum 6 and left operculum. This pattern aligns with previous research on motor skill learning in badminton and drumming, suggesting a generalizable effect of skill acquisition on brain functional reorganization (Amad et al., 2017; Shao et al., 2019). Notably, the observed changes were concentrated within the temporal, frontal, and cerebellar regions, with particular emphasis on the pSTG and toMTG within the temporal cortex. These regions play critical roles in visual processing, perception, and spatial processing, all of which are fundamental for skill learning (Goodale et al., 1991; Jo et al., 2019). The strengthened connectivity between the orbitofrontal cortex and temporal lobe regions is likely driven by the visual information about the ball and its surroundings that the OFC receives from these areas (Rolls, 2004; Kable and Glimcher, 2009; Rolls and Treves, 2011). Notably, studies like Gazzaley et al. (2007) have shown that prefrontal-visual connectivity strengthens during tasks requiring focused attention, such as encoding facial or scene stimuli, suggesting its potential role in learning visual information. Therefore, we hypothesize that the focused attention required to track the dynamic movements of the ball and its environment during football juggling drives the increased functional connectivity between the OFC and temporal lobe regions.

Existing research has established a strong link between cerebellar areas 4–5 and sensorimotor processing (Grodd et al., 2001; Habas et al., 2009; Sang et al., 2012; Stoodley et al., 2012; Wang et al., 2016). In addition, previous investigators found that cerebellum 4 is associated with foot action. For example, in Grodd et al.'s study, participants were asked to perform several movement tasks with their eyes closed. They found that flexing or extending the foot activated cerebellum 4 (2001). Cerebellum 6 has been demonstrated to be involved in motor coordination in complex movements, such as visually guided movements and sequential movements (Schlerf et al., 2010). Schlerf et al. (2010) study demonstrated this by observing cerebellum 6 activation only during complex finger and toe movement sequences, not simpler simultaneous movements. Indeed, juggling is a highly complex task that requires constant switching between feet. This complexity likely explains why football juggling learning enhances the connectivity between the temporal lobe regions and the cerebellum. Furthermore, we found that increased functional connectivity between the temporal lobe and the subcortical regions (specifically, the cerebellum) was unique to new motor skill learning. This aligns with the research of Spampinato et al., who found no changes in connectivity between the cortex and cerebellum when performing a familiar motor task but significant changes when learning a new one. This finding suggests that new motor skill learning requires more extensive communication between the cortex and cerebellar regions (Spampinato and Celnik, 2017, 2021). In this paper, football juggling learning presents a new motor challenge for participants with no experience in football training. This novel challenge further strengthens the observed connectivity of the cerebellum and temporal lobes.

Beyond extensive increases in connected cortical regions within the prefrontal lobes, temporal lobes, and cerebellum, our study revealed additional heightened connectivity between the temporal and occipital lobes. Notably, the occipital lobes are integral to the ventral visual pathway and crucial for human object perception, recognition, and encoding of spatial relationships within scenes (Grill-Spector and Weiner, 2014). Prior drumming research suggests that 8 weeks of training can enhance functional connectivity between the temporal and occipital lobes, potentially key to multisensory learning. Given that football juggling demands complex multimodal skills, including limb motor activity heavily reliant on multisensory feedback (Amad et al., 2017), we speculate that this feedback bolsters functional connectivity between the temporal and occipital lobes. Our findings consistently indicate that football juggling learning has the potential to induce increased functional connectivity. However, research on brain functional networks in gymnasts and non-athletes suggests extensive motor skill training can lead to reduced connectivity within specific networks, such as the basal ganglia and left/right fronto-parietal networks (Huang et al., 2018). We hypothesize that during the early stages of motor skill learning, unfamiliarity with movements necessitates strengthened connectivity and resource integration between brain regions to support action execution. As proficiency increases, redundant and irrelevant connections are pruned, establishing more efficient neural pathways. Our study focused on completing football juggling learning (achieving 35 continuous juggles). While this benchmark reflects a notable level of proficiency, it is important to acknowledge that it may not directly predict the attainment of fully automated juggling capabilities. Future research could extend the training duration to further explore the evolving trends of brain functional connectivity during motor skill learning.

Finally, our study revealed a significant correlation between enhanced functional connectivity and improved shifting performance, echoing findings from earlier exercise interventions. Similar to Li et al. (2014), who reported a link between increased resting-state connectivity and enhanced cognitive behavior after a multimodal intervention, we observed a predominant association between improved shifting performance and enhanced functional connectivity of the right pSTG to the left cerebellum 6. Notably, both the pSTG and cerebellum 6 are implicated not only in motor skill learning but also in EF (Pi et al., 2019; Wang et al., 2020; Mcdougle et al., 2022; Yang et al., 2022). Given the cerebellum's crucial role in the interconnected cortical circuit underlying EF development, we propose that the observed improvement in EF due to football juggling learning can be attributed to the strengthened functional connectivity between the pSTG and cerebellum 6.

This study sheds light on the potential link between motor skill learning and executive function, specifically suggesting that football juggling may enhance EF through brain connectivity reorganization. However, several limitations deserve further consideration. First, our investigation solely focused on football juggling, leaving the impact of other motor skills on EF unexplored. Future studies should examine the effects of diverse motor skills on EF to broaden our understanding. Second, our data collection was limited to pre- and post-football juggling time points. Future research should incorporate data across various motor learning stages to elucidate the nuanced effects of different learning phases on EF. Finally, our control group lacked a non-motor skill learning task during the football juggling learning period, limiting our ability to control for non-specific learning effects. Additionally, potential confounding variables such as end-of-term stress and aerobic fitness were not accounted for. Acknowledging these limitations, future research should prioritize incorporating control tasks and meticulously controlling for potential confounding variables to further validate and refine our understanding of the relationship between motor skill learning and EF.

## 5 Conclusion

This longitudinal study provides evidence for the efficacy of football juggling in boosting both performance in executive functions and functional connectivity within the temporal, frontal, and cerebellar brain regions. Notably, the enhanced functional connectivity between the right posterior superior temporal gyrus and the cerebellum may underpin the observed improvements in executive function. These findings highlight the significance of motor skill learning as a potential avenue for cognitive enhancement and brain plasticity. They pave the way for future research exploring the potential of motor skill learning interventions to improve cognitive abilities and induce neuroplastic changes. It is noteworthy that the control group in this study also exhibited significant improvements in executive functions, although to a lesser extent than the FTL group. This observation necessitates a cautious interpretation of the results and underscores the need for further research to explore the underlying mechanisms at play.

## Data availability statement

The raw data supporting the conclusions of this article will be made available by the authors, without undue reservation.

## Ethics statement

The studies involving humans were approved by Institutional Review Board of the Affiliated Hospital of Yangzhou University (2017-YKL045-01). The studies were conducted in accordance with the local legislation and institutional requirements. The participants provided their written informed consent to participate in this study.

## Author contributions

XD: Conceptualization, Data curation, Investigation, Methodology, Visualization, Writing—original draft. XG: Data curation, Investigation, Methodology, Visualization, Writing—original draft. SK: Writing—review & editing. LZ: Writing—review & editing. DC: Writing—review & editing. ZS: Writing—review & editing. YS: Writing—review & editing. AC: Conceptualization, Writing—review & editing.
